# Topics of Public Interest

**Published:** 1913-07

**Authors:** 


### TOPICS OF PUBLIC INTEREST.

---

Chautauqua County Tuberculosis Hospital Endowed. Mrs. Elizabeth M. Newton, a former resident of Fredonia, who died in California about the middle of May, has left $150,000 for this purpose. She also left $6,000 to endow a bed in the Brooks Memorial Hospital of Dunkirk for poor patients from Fredonia, and other sums for charitable purposes.

---

Graduation and License of Physicians. A noteworthy difference exists between the number of graduates and of licenses issued. In 1912 the former amounted to 4,483, the number being about the same for 1911; 6,723 licenses were issued, 5,466 by examination, 116 by registration under exemption laws, 1,141 by reciprocity. Six thousand one hundred physicians were examined, the apparent discrepancy being due to the proportion of failures. Of these 6,100, 5,287 were recent graduates, 1908-1912; 843 of longer standing. These figures indicate a considerable number of removals in the attempt to find a satisfactory location, not to mention changes of location in any one state.

The Crouse-Irving Hospital, a private institution of Syracuse, has been incorporated and its scope and capacity will be enlarged.

---

| Total resistance of body assumed to be | Results of brief exposure to passage of alternating current at a pressure of | | |
|---|---|---|---|
| | 100 volts | 1,000 volts | 10,000 volts |
| Very low, with good contact, 1,000 ohms | Certain death, slight burns | Probable death, marked burns | Survival, burns and other sequelae very severe |
| Higher, about 10,000 ohms | Painful shock, but no injury. | Certain death, slight burns | Probable death, severe burns |
| High, with bad contacts, 100,000 ohms | Scarcely felt | Painful shock, but no severe injury | Certain death, burns slight if resistance remains high |

—Jex Blake.

---

Proportion of Immigrants Debarred in Last Five Years For Medical Reasons.

| Year | Total Immigration | Debarred per 100,000 for Following Reasons: Mental Defects | Epilepsy and Insanity | Loathsome or Dangerous Contagious Disease |
|---|---|---|---|---|
| 1908 | 782,870 | 23.7 | 23.5 | 371 |
| 1909 | 751,786 | 24.1 | 22.2 | 317 |
| 1910 | 1,041,570 | 17.4 | 19.0 | 300 |
| 1911 | 878,587 | 18.8 | 16.6 | 325 |
| 1912 | 838,172 | 19.7 | 16.0 | 208 |

Note.—Compiled from annual report of Commissioner General of Immigration for fiscal year 1912.

---

Hospital For the Tonawandas. Business men have offered $40,000 for the erection of hospital on Christiana street, North Tonawanda, provided the city will provide for its maintenance. Efforts have been made for twenty years in this direction and we trust that the Twin Cities, which ought to be one, will recognize the need of co-operation to insure economic concentration of management.

---

Lockport Quarantine Hospital. The Board of Health has adopted a second resolution, urging the Common Council to provide such an institution, Buffalo having notified that Lockport patients can no longer be accommodated.

### Statistics of Physicians.

| States | Total 1 Physicians | 1 Allopaths | 2 Homeopaths | 3 Eclectics | 4 Osteopaths | 5 Unclassified |
|---|---|---|---|---|---|---|
| Alabama ........ | 2,418 | 2,332 | 12 | 50 | 12 | 24 |
| Arizona ......... | 247 | 227 | 14 | 4 | 4 | 2 |
| Arkansas ....... | 2, 96 | 2,505 | 36 | 30 | 23 | 25 |
| California ...... | 4,796 | 3,967 | 408 | 374 | 432 | 47 |
| Colorado ........ | 1,772 | 1,516 | 171 | 68 | 127 | 17 |
| Connecticut ..... | 1,564 | 1,315 | 151 | 83 | 25 | 15 |
| Delaware ....... | 246 | 205 | 33 | 6 | 2 | 2 |
| Dist. of Columbia | 1,350 | 1,250 | 78 | 9 | 23 | 13 |
| Florida ......... | 974 | 908 | 30 | 27 | 20 | 9 |
| Georgia ......... | 3,022 | 2,633 | 25 | 334 | 47 | 30 |
| Idaho ........... | 420 | 374 | 17 | 25 | 40 | 4 |
| Illinois .......... | 9,988 | 8,094 | 1,112 | 683 | 336 | 99 |
| Indiana ......... | 4,984 | 4,077 | 297 | 557 | 82 | 49 |
| Iowa ............ | 3,653 | 3,005 | 387 | 225 | 136 | 36 |
| Kansas .......... | 2,688 | 2,155 | 208 | 299 | 152 | 26 |
| Kentucky ........ | 3,601 | 3,301 | 134 | 130 | 64 | 36 |
| Louisiana ........ | 1,930 | 1,857 | 30 | 24 | 15 | 19 |
| Maine ........... | 1,176 | 1,045 | 97 | 23 | 18 | 11 |
| Maryland ....... | 1,972 | 1,800 | 141 | 12 | 14 | 19 |
| Massachusetts ... | 5,648 | 4,858 | 635 | 99 | 160 | 56 |
| Michigan ........ | 4,104 | 3,243 | 582 | 238 | 123 | 41 |
| Minnesota ....... | 2,262 | 1,987 | 196 | 57 | 106 | 22 |
| Mississippi ...... | 2,009 | 1,960 | 3 | 26 | 20 | 20 |
| Missouri ......... | 6,037 | 5,114 | 319 | 544 | 296 | 60 |
| Montana ........ | 512 | 485 | 25 | 7 | 40 | 5 |
| Nebraska ........ | 1,796 | 1,349 | 126 | 304 | 101 | 17 |
| Nevada .......... | 144 | 131 | 5 | 6 | 5 | 2 |
| New Hampshire.. | 704 | 614 | 64 | 19 | 9 | 7 |
| New Jersey ..... | 2,884 | 2,456 | 345 | 55 | 125 | 28 |
| New Mexico ..... | 430 | 395 | 18 | 13 | 21 | 4 |
| New York ....... | 14,815 | 12,805 | 1,305 | 557 | 288 | 148 |
| North Carolina .. | 1,849 | 1,813 | 8 | 10 | 23 | 18 |
| North Dakota ... | 594 | 563 | 15 | 11 | 24 | 5 |
| Ohio ............. | 7,513 | 5,786 | 906 | 746 | 160 | 75 |
| Oklahoma ....... | 2,620 | 2,337 | 62 | 195 | 88 | 26 |
| Oregon .......... | 1,041 | 933 | 46 | 52 | 98 | 10 |
| Pennsylvania .... | 11,345 | 9,812 | 1,153 | 267 | 272 | 113 |
| Rhode Island .... | 751 | 686 | 57 | 1 | 17 | 7 |
| South Carolina ... | 1,275 | 1,254 | 4 | 5 | 12 | 12 |
| South Dakota .... | 651 | 560 | 37 | 48 | 32 | 6 |
| Tennessee ....... | 3,338 | 3,128 | 28 | 149 | 52 | 33 |
| Texas ........... | 5,888 | 5,479 | 82 | 269 | 120 | 58 |
| Utah ............ | 427 | 395 | 14 | 14 | 12 | 4 |
| Vermont ........ | 670 | 592 | 57 | 24 | 12 | 6 |
| Virginia ....... | 2,359 | 2,283 | 35 | 18 | 16 | 23 |
| Washington ..... | 1,630 | 1,481 | 73 | 60 | 136 | 16 |
| West Virginia ... | 1,639 | 1,492 | 28 | 103 | 17 | 16 |
| Wisconsin ....... | 2,652 | 2,289 | 224 | 113 | 50 | 26 |
| Wyoming ........ | 235 | 216 | 10 | 7 | 7 | 2 |
| Total ......... | 137,199 | 118,666 | 9,954 | 7,330 | 3,994 | 1,349 |

(1) A. M. A. Medical Directory, (2) W. A. Dewey, M. D., of Ann Arbor, Mich., estimate, (3) is a little over an exact count of the new card index of chief organizer Dr. Mundy, (4) *American Journal of Osteopathy*.—Assembled Statistics by *The N. E. M. A. Quarterly*.

Railroad Accidents. The Interstate Commerce Commission reports, for the fiscal year ending late in 1912, 169,538 injuries, 19,379 more than for the previous year, and 10,580 deaths, 394 more than for the last year. Approximately 1,500,000 deaths occur annually from all causes, so that about two-thirds of 1 per cent. of the annual death rate is directly due to railroads. Deaths due indirectly to injuries received in railroad accidents. probably bring the proportion above 1 per cent. Railroad fatalities have increased by about 50 per cent. since 1909. About 4 per cent. of all deaths are due to violence, the honors being about equally divided between suicide and railroads. Railroad transportation kills about one and a half to twice as many as all other forms of transportation. Germany, Austria, Russia and France injured 2,494 and killed 394 by railroads last year.

---

County Tuberculosis Hospitals. In our May issue we noted the opposition of Dr. John A. Rafter, Mayor of North Tonawanda, to the establishment of a Tuberculosis Hospital for Niagara County, on the ground of unsuitability of climate. Without taking sides as to the value of climate in the treatment of tuberculosis, except to the extent of calling attention to the various changes of professional opinion observed in the last thirty years regarding the optimum climate and citing personal and other experience that tuberculosis could be recovered from in the native environment, even if not especially favorable, we expressed the opinion that Dr. Rafter's opposition would serve a valuable end in bringing the discussion regarding county (or, for practical purposes, local) hospitals to a focus on the therapeutic point involved. Previously the problem has been mainly economic and with regard to securing necessary legislation.

The State Charities Aid Association, in its May *News*, publishes a considerable series of letters, all favorable to the County Hospital, or, at any rate, to some easily accessible institution. These we reproduce, as well as the letter from Dr. Hawes, not published in the *News*.

I hope to see many special hospitals thruout New York State to care for tuberculosis patients near their homes; it is both practicable and advisable to have such hospitals.

Simon Flexner, M. D.,
New York.

(Director Rockefeller Institute for Medical Research.)

Most important function of tuberculosis hospitals is to furnish institutions where advanced or moderately advanced consumptives can be rendered no longer dangerous to communities. Accessibility is therefore first consideration.

HERMANN M. BIGGS, M. D.,
New York.

(General Medical Officer New York City Department of Health —Chairman Governor Sulzer's Public Health Commission.)

Our best hospital for consumptives is within Boston limits at Mattapan. Lakeville, North Reading Hospitals all at low altitude. Care of advanced cases important for prevention.

VINCENT Y. BOWDITCH, M. D.,
Boston.

(Former President of the National Association for the Study and Prevention of Tuberculosis.)

Most important point in tuberculosis hospital provision is location close to community to be served. Climate anywhere in New York State is very favorable for treatment.

LIVINGSTON FARRAND, M. D.,
New York.

(Executive Secretary National Association for the Study and Prevention of Tuberculosis.)

It is my firm conviction, based upon years of experience, that in the location of a hospital for the tuberculous, accessibility, convenience and economy are more important than any special climate and should be considered first.

S. A. KNOPF, M. D.,
New York.

(International Authority on Tuberculosis.)

Tuberculosis hospitals should be near center of population; dangerous cases will not, and far advanced cases cannot go far from home.

E. L. TRUDEAU, M. D.,
Saranac Lake.

(Pioneer of Outdoor Treatment of Tuberculosis in the United States.)

Such institutions should be and are usually near homes of patients, elevated, if possible, in as good situation as possible, but always accessible regardless of climate.

E. R. BALDWIN, M. D.,
Saranac Lake.

(Leading Tuberculosis Expert, Director Harriman Research Laboratory and Member of Governor Sulzer's Special Public Health Commission.)

Climatic conditions of far less importance than proximity to center of population in county. Would strongly urge hospital to be built within five cent carfare of large city.

LAWRASON BROWN, M. D.,
Saranac Lake.

(Former Superintendent Adirondack Cottage Sanatorium, Saranac Lake.)

County hospital should be for advanced cases and located near homes of sick and relatives; more important than ideas of climate; open air treatment will be successful anyway if proper management and maintenance. JOHN H. PRYOR, M. D.,
Buffalo, N. Y.

Governor Sulzer's Public Health Commission recently reported to Governor, among other things, that county tuberculosis hospitals should be established in counties not now provided therewith. Medical authorities regard accessibility of far greater consequence than climate. ANSLEY WILCOX.

(Member Sulzer Public Health Commission and President of the Charity Organization Society of Buffalo.)

The wisdom of having the sanatorium accessible to centers of population has been demonstrated by the growth of Iola, Monroe County's tuberculosis sanatorium, adjacent to the city line of Rochester. In two and a half years it grew from ten patients to one hundred and twenty-seven with a constantly increasing waiting list and the hearty support of the local physicians.

MONTGOMERY S. LEARY,
For Board of Managers.

(Superintendent Monroe County Tuberculosis Hospital.)

The great local problem is caring for those who could never and would never go to a distance for treatment. To segregate the advanced cases is to protect the public from infection; each county must perfect measures to protect its own people.

JOHN F. W. WHITBECK, M. D.,
Rochester.

(President Medical Society of the State of New York.)

Treatment of tuberculosis cases very important as a preventive measure. Location of tuberculosis hospital close to centers from which patients will come, especially advanced cases, is far more important than the matter of climate.

FRANCIS E. FRONCZAK, M. D.

(Health Commissioner, City of Buffalo.)

The Commonwealth of Massachusetts,
Trustees of Hospitals for Consumptives,
3 Joy Street, Boston, Mass.

May 16th, 1913.

Mr. George J. Nelbach,
State Charities Aid Association,
105 East 22d St., New York, N. Y.

Dear Sir:

I take pleasure in answering the questions you have asked me to the best of my ability.

1. The State of Massachusetts did treat tuberculosis at first at a state problem. In the days that the State Sanatorium at Rutland was first established our one idea was to endeavor to cure the incipient cases.

2. As time went on we have certainly radically changed our views on the subject until those of us who are acquainted with the facts regard tuberculosis primarily as a local problem due to local causes. This change of opinion is due to the fact that we realize more and more that the advanced consumptive has every right to be kept in hospitals near his family and friends where he can be seen at frequent intervals. Of course, this does not mean in the slightest that we are going to do away with our State Sanatoria for the early and curable cases, as, in fact, we are constantly increasing all four State institutions.

3. Under the provision of the act passed in 1911 and modified in 1912, but not by any means as the result of these acts, about sixteen or seventeen of the towns and cities in this State of ten thousand inhabitants or over have provided themselves, or were already provided, with local tuberculosis hospitals, while about an equal number are either actually constructing, or are actively planning, such hospitals.

Finally, I would say that I have no sympathy with those who go to an extreme in this matter either in demanding that the work must be done entirely by the State or that it must be done entirely by local forces. There must be a wise and practical combination of both forces acting under a broad co-ordinated scheme.

Yours truly,
(Signed) John B. Hawes, 2d,
Secretary.

In fairness to those of Dr. Rafter's opinion, it should be stated that the idea that climatic conditions have an important bearing on the treatment of tuberculosis is still strongly urged by many authorities, the argument being based both on empiricism and scientific consideration of the effects of sun-light, altitude, degree of humidity, etc. We have always understood that the Saranac Lake authorities, considered their location as of direct climatic value and not as a matter of accidental convenience, while the J. N. Adam Hospital was deliberately located in Cattaraugus Co. at an altitude almost as great as that of Raybrook, accessibility being sacrificed for the sake of securing this advantage. It will also be noted that several of the opinions quoted apply especially to the treatment of advanced and incurable cases for which climate is admittedly a minor consideration and for which the institution of special hospitals depends mainly upon administrative details and the question as to the communicability of the disease. In regard to the last question, the same number of the *News* contains an article by Dr. Trudeau, entitled "Sanatoria not a Menace," alluding to the alleged danger to neighboring population. Regarding the accessibility of a hospital, it should be borne in mind that the problem for a county like Niagara is quite different to that presented to counties of absolutely much greater population and with 90 per cent. or more of the population concentrated in one city. Niagara County has a total population of 92,000, of which 30,000 is in Niagara Falls, 18,000 in Lockport and 12,000 in North Tonawanda, the rest scattered. So far as climate is concerned, the main point to be considered is altitude, although strictly local items, as nature of soil, swamps, factory fumes, dust, exposure to wind, etc., would present themselves. We are inclined to concede that the problem of selecting an available site, with reference to economy, salubrity and accessibility might present many unforeseen difficulties. In regard to aitltude, Niagara County is divided pretty sharply by the Niagara escarpment into plains, one slightly above the Lake Ontario level, the other averaging somewhat lower that the Lake Erie level. With the obvious qualification that there are individual exceptions, especially along the boundary, it may be said that the higher altitudes of Niagara County are lower than the lower altitudes of Erie County. With the recent highly approved action of Erie County of going beyond its own hilly region to secure a better site in Cattaraugus County and the existence of a considerable weight of professional opinion favoring moderately high or even mile-high altitudes, and with the successful campaign for state care of the tuberculous in specially selected, elevated regions, still a matter of recent history, it seems to us that a judicial attitude should be assumed toward Dr. Rafter's contention.

To prevent misunderstanding, we may add that we do not personally regard altitude or any other climatic factor consistent with general salubrity as essential in the treatment of tuberculosis; on the contrary, we rather favor the idea that the average—

though not every—patient will do best in the climate to which he is accustomed. We hold also that every person, with tuberculosis or any other lesion, who cannot command adequate private means for proper treatment, should be publicly assisted. Whether this assistance is given by the county, municipality, state or nation, is to be decided according to practical considerations only indirectly related to medicine. We are not inclined to make the name County Tuberculosis Hospital a shibboleth since the term county stands for very different conceptions of wealth, population, density of population, prevalence of the disease, proportion of dependent population, availability of salubrious sites, etc.—not to mention that the rather sudden drop from the authoritative opinion regarding high altitudes has rather stunned our reasoning faculties on this point. The fact mentioned by Assistant Secretary George J. Nelbach of the S. C. A. A., that other states have blindly copied the New York law, even to "mistake in phraseology and pronounciation," confirms our opinion, expressed in the May issue, that Dr. Rafter, in his role of a doubting Thomas, has done a real service to the cause by interposing a temporary obstacle.

---

EXAMINATION OF CANDIDATES FOR ASSISTANT SURGEON. The Public Health Service announces examinations in Washington, Boston, New Orleans and San Francisco, July 7 and August 4. The initial salary is $2000 with quarters, or commutation at $30 per month. Appointments are permanent and promotions to be expected gradually lead to a salary of about $6000. This is in many ways the most desirable government medical service.

---

THE CASE OF THE ROME INFIRMARY DOCTORS. Last year the Oneida County Medical Association expelled four physicians practicing under this title, and, as we understand, advertising their services to the laity. The expelled members appealed to the State Society and then secured a temporary injunction restraining the County Association from pressing charges. Judge Pardon C. Williams, Referee, has handed down the following opinion. On account of the general principles involved, it becomes a most valuable precedent in cases of ethics, all the more so because it does not decide the actual case presented. In this connection we may allude to the opinion of a physician on the staff of a somewhat similar advertising institution. He held that so long as he belonged to the institution, it would be unfair for him to accept membership in the regular medical organizations, even though others, similarly circumstanced had been admitted. He declared that he was performing his routine medical duties conscientiously, according to the best of his ability, and that he intended, as soon as he was financially able, to enter private practice, when he hoped he would be admitted to the regular medical organization of the county and state. And we grasped his hand and told him he had the true conception of ethics.

"I do not pass upon or find as to the question of the plaintiff's guilt or innocence of charges against them.

"I decide as a matter of law:

"(1) The plaintiff having joined the defendant society and the state society subjects himself to their constitutions and by-laws with reference to the preferring of charges against their members and the investigation thereof and discipline therefor.

"(2) The censors of the defendant society having investigated the charges preferred against the plaintiff and reported thereon to the defendant society this court should not at this time inquire into such investigation and report or pass upon the question as to the correctness thereof.

"(3) The state society upon the plaintiff's appeal, having determined that the charges preferred against the plaintiff were within the contemplation of the by-laws of the defendant society and proper subjects for investigation and discipline, this court should not inquire or pass upon these questions.

"(4) This court should not restrain the defendant society from acting on the censor's report or from any particular action thereon and should not now inquire into or pass upon what action the defendant should take on the said report.

"(5) The defendant should have judgment dismissing the plaintiff's complaint and dissolving the temporary injunction with costs, and judgment is ordered accordingly."

---

CONSERVATIVE EFFECT OF INCREASED MATRICULATION REQUIREMENTS. The Annual Announcement of the University of Colorado contains the following significant statistics. Graduates, 1912, 39; enrollment, 1913, 53; 1914, 15; 1915, 9; 1916, 15. We take it that the increase in the class of 1913 was due to the endeavor to anticipate the more stringent requirement of two years of college work and that the sudden drop was due to the inforcement of the higher standard. There are two or three obvious morals to be drawn. Inadequately prepared men must not merely be shifted from one state to another, but every state and, especially, the wealthier eastern communities and the stronger colleges have a plain duty of raising their standards to the level of the western states. The marked reduction in attendance will act but very gradually and slowly, to restore the medical profession to an economic equilibrium. Medical education can no longer be carried on, generally, at a profit or even on a self-supporting basis but public or private philanthropy must assist as well as control the medical colleges. We do not believe in dollar diplomacy in this regard. A school should survive, not as a matter of financial fitness but of fitness according to higher ethical standards.

---

New York State Department of Labor, Bureau of Statistics and Information.—Official Notice to Physicians.—By Chapter 145, Laws of 1913, two additional industrial diseases are required to be reported by physicians to this Department, viz, *poisoning by brass* or *wood alcohol.* The eight diseases which must now be reported are: Lead poisoning (plumbism), Phosphorus poisoning, Arsenic poisoning, Brass poisoning (brass founder's ague), Wood Alcohol poisoning, Mercury poisoning, Anthrax, Compressed air illness (caisson disease).

---

Typhoid and Water Supply. Attention is called to the very valuable special article in the *Jour. of the A. M. A.,* page 1702, May 31. Buffalo had a death rate of 11.4 per 100,000 for 1912; 25.1 for 1911; average 22.8 for 1906-10. The figures for Rochester were, respectively, 11.8, 10.3, 12.4; for Syracuse 16.8, 15.7, 15.7; for Albany, 17.7, 17.8, 17.5 (and will probably be much higher this year on account of the inundation of the filtration plant); for New York 9.8, 11.1, 13.8. According to the census of 1910 there were just fifty cities of over 100,000 population, aggregating 22 per cent. of the entire population of the country. Until recently, 25:100,000 typhoid mortality was considered fairly low and Pittsburgh averaged 74.3 from 1906 to 1910, but this has dropped to 12.7 since filtering the water for Pittsburgh proper, in fact, for this part of Greater Pittsburgh, the mortality for 1912 was only 5.9. The lowest rate has been maintained by Cambridge, Mass., 9.8 for 1906-10 down to 2.8 for 1912. Buffalo is, we believe, the only large city of the U. S. which maintains a fairly low rate on a supply of "raw" water and an unguarded water shed. Criticism of this statement is requested, but read it carefully. We believe that the actual, resident typhoid mortality of Buffalo is not much over half the amount stated, although a similar statement may perhaps be required for various other cities, and we believe that a good deal of the resident mortality is acquired from water drunk outside the city. A very careful study of the question of where the typhoid cases in Buffalo live and what water they have drunk, or how they have been exposed to other sources of infection, should be made before undertaking any expensive method of protecting the water supply.

Husted Mill Explosion. During the afternoon of June 25, an explosion took place in the Husted Mill of Buffalo. One man was blown 500 feet in the air; the engineer and fireman of a passing locomotive were fatally injured; two others were killed either by fire or crushing. More than sixty men required hospital treatment for burns and traumatisms, and the total deaths will probably reach twenty-five. The building itself was fire proof, but, as in Baltimore, Philadelphia and New York, serious fires may occur in fire-proof buildings. As a layman well expressed it regarding the last, in which so many girls were burned to death, "it was like a fire in a stove." Fires of the present nature are, fortunately, rare and those first noted, a quarter of a century ago, were regarded as mysterious. It has, however, been demonstrated that grain dust of various kinds, in a state of fine subdivision and concentration becomes not only theoretically combustible by reason of its carbohydrate but actually inflammable and explosive—probably in analogy to the increase of combustibility and explosiveness of gasoline, kerosene, etc., when sprayed by a carburetor. It would be worth while for those interested in industrial prophylaxis to pay special attention to the contents of fire-proof buildings and to investigate and act upon the contention that dust-explosions are impossible if the air does not become surcharged with dust.

